# Prevalence and Risk Factors for Resistant Hypertension: Cross-Sectional Study From a Tertiary Care Referral Hospital in South India

**DOI:** 10.7759/cureus.18779

**Published:** 2021-10-14

**Authors:** Rima Mahapatra, Anupriya Kaliyappan, Palanivel Chinnakali, Nandeesha Hanumanthappa, Ramkumar Govindarajalou, Chanaveerappa Bammigatti

**Affiliations:** 1 Medicine, Jawaharlal Institute of Postgraduate Medical Education and Research, Puducherry, IND; 2 Preventive Medicine, Jawaharlal Institute of Postgraduate Medical Education and Research, Puducherry, IND; 3 Biochemistry, Jawaharlal Institute of Postgraduate Medical Education and Research, Puducherry, IND; 4 Radiodiagnosis, Jawaharlal Institute of Postgraduate Medical Education and Research, Puducherry, IND

**Keywords:** resistant hypertension, prevalence, non-adherence, therapeutic inertia, south india, risk factors, secondary hypertension, uncontrolled diabetes, obstructive sleep apnoea

## Abstract

Background

Around 10% patients with hypertension have resistant hypertension (RH). Older age, Black race, obesity, diabetes mellitus (DM) and chronic kidney disease (CKD) are the common risk factors for RH. The present study was done to find out the prevalence and factors associated with RH.

Methods

This cross-sectional study was done between December 2018 and February 2020. Adult patients registered with the hypertension clinic and on care for more than three months were included in the study. History was noted and blood pressure (BP) was measured using standard precautions. The patients were divided into two groups - resistant and non-resistant hypertension. Chi-square test was done to check the significance of the differences between the two groups. Binary logistic regression was done for the risk factors with a p-value < 0.2 in the Chi-square test.

Results

A total of 275 patients were included. The mean age was 56 ± 10 years and 61% were females. The mean duration of hypertension was 7 ± 5 years; 77% of patients were overweight or obese. A family history of hypertension was present in 30% and 18% had diabetes mellitus. History suggestive of secondary hypertension was present in 13%. BP was controlled (<130/80 mm of hg) in 145 (53%), uncontrolled in 130 (47%) and resistant hypertension was seen in 31 [(11%) 95% CI 8-16%] patients. Duration of hypertension, obesity, and elevated fasting blood sugar were significantly associated with RH.

Conclusions

RH was found in 11% of hypertensive patients. Longer duration of hypertension, obesity, and higher fasting blood glucose were associated with RH.

## Introduction

High blood pressure (BP) has a graded association with increased cardiovascular disease (CVD) risk [1]. Twenty mm Hg higher systolic BP and ten mm Hg higher diastolic pressure each is associated with twice the risk of dying from stroke, heart disease, or other vascular diseases [2]. Despite this, many are undiagnosed and diagnosed hypertensives also are not having controlled blood pressure even in the developed world [3]. Various factors which may lead to inadequate control of BP are nonadherence to treatment regimens, inappropriate dietary habits, failure of doctors to intensify the treatment regimens (therapeutic inertia), white coat effect (elevated office BP but normal out-of-office BP), or true treatment-resistant hypertension.

Resistant hypertension (RH) is defined as elevated BP above-goal despite the concurrent use of three antihypertensive drug classes, commonly including a long-acting calcium channel blocker, a blocker of the renin-angiotensin system (angiotensin-converting enzyme inhibitor or angiotensin receptor blocker), and a diuretic. The antihypertensive drugs should be administered at maximum or maximally tolerated daily doses. RH also includes patients whose BP achieves target values on ≥ four antihypertensive medications. [4].

Meta-analysis of data from 3.2 million patients has estimated that the global prevalence of true resistant hypertension is 10%, which was lower than the prevalence of apparent treatment-resistant hypertension (when pseudo-resistant hypertension is not excluded) [5].

Common risk factors for resistant hypertension include older age, obesity, chronic kidney disease (CKD), Black race, and diabetes mellitus (DM). All the available studies so far have taken BP control target of < 140/90 mmHg but the American Heart Association revised the control target to < 130/80 in 2017 [6]. It is estimated that the prevalence of RH with the new control target would be 4% higher as compared to the older control target.

We undertook this study to find out the prevalence of RH among patients attending a hypertension clinic at a tertiary care referral hospital in South India and also studied the factors associated with RH using the newer control target as there were no previous such studies in this region. 

## Materials and methods

Setting

This was a cross-sectional, analytical study conducted in the Hypertension Clinic of Jawaharlal Institute of Postgraduate Medical Education and Research (JIPMER), Puducherry, a tertiary care referral hospital in South India catering to patients mainly from Puducherry, neighbouring districts of Tamil Nadu, Kerala, and Andhra Pradesh, between December 2018 and February 2020. The hypertension clinic at JIPMER runs every Tuesday between 14.00 and 16.30 hours and around 100 to 150 patients attend the clinic every week. Patients are given free medicines every month during follow-up visits. The study was approved by the Institute Ethics Committee (JIP/IEC/2018/0187).

Participants

Adult (> 18 years of age) patients registered with hypertension clinic and on treatment for more than three months were included in the study. There were no exclusion criteria used for our study. A list of patients attending the clinic for a two-month period was prepared and consecutive patients were included from the line list till the sample size was achieved.

Sample size calculation

Assuming the proportion of patients with resistant hypertension as 20% based on a study done in a similar setting as ours in Sri Lanka [7] with absolute precision of 5% and 95% confidence level, the calculated sample size was 246. Considering a non-response rate of 10%, we included 275 hypertensive patients for the study. Sample size was calculated using OpenEpi, version 3.03 (www.OpenEpi.com).

Procedure

Eligible patients were enrolled in the study after taking written informed consent. Demographic details (age, sex, occupation), lifestyle-related information (smoking, excess alcohol and excess salt consumption, physical inactivity), treatment-related information (age at diagnosis of hypertension, number, and doses of antihypertensive medications, adherence to medication, other co-morbidities, intake of other medications such as nonsteroidal anti-inflammatory drugs (NSAIDs), oral contraceptive pills, cyclosporine, erythropoietin, etc.), family history of hypertension and features suggestive of secondary hypertension (erratic sleep, excessive snoring, daytime sleepiness, weight gain, change of appearance, i.e., moon face, proximal muscle weakness and episodes of palpitation, sweating and tremors) were noted in a predesigned proforma. The presence of diabetes mellitus, ischemic heart disease (IHD), chronic kidney disease, dyslipidemia, and thyroid disorders were confirmed from the previous records of the patients.

Measurement of BP

Standard precautions were used while measuring blood pressure. Participants were advised to empty the bladder if needed and asked to sit relaxed with feet on the floor and arm and back supported for five minutes. No smoking, exercise, or intake of caffeine within 30 minutes preceding the measurement was ensured. Arm circumference was measured and appropriate cuff size was used accordingly. BP was measured with a mercury sphygmomanometer in both arms and in the standing position to check for orthostatic hypotension. BP was rechecked after few minutes in the arm with higher recording. The second reading was taken as the actual blood pressure of the patient.

Other relevant physical examinations and investigations were done to identify secondary hypertension and target organ damage. Adherence to antihypertensive medications was assessed using the Morisky Green Levine adherence scale [8]. A score with ≥3 out of 4 was considered as non-adherence.

Definitions

Uncontrolled hypertension was defined as blood pressure ≥ 130/80 mm of Hg. Uncontrolled resistant hypertension was defined as blood pressure ≥ 130/80 mm of Hg on three or more antihypertensive drugs with optimal doses including a diuretic. Controlled resistant hypertension was defined as blood pressure < 130/80 on more than three antihypertensive drugs with maximal/maximally tolerated doses including a diuretic. Excess alcohol consumption included binge drinking, heavy drinking, and any drinking below 21 years of age. Physical activity was defined as 30 minutes of aerobic exercise five times per week for a total of 150 minutes per week. BMI was categorized according to 2004 WHO classification for the Asian population. Overweight is defined as BMI of 23-24.9 kg/m2 and obese is defined as BMI ≥ 25 kg/m2.

Estimation of salt intake

The following formula was used for the estimation of 24-hour urinary sodium excretion from spot urinary sodium and creatinine values [9].

$PR\, Na = 21.98 \times (\frac{Na\, spot}{Cr\, spot \times 113.1} \times PR\, Cr) \times 0.392$

[Where PR Na = predicted 24-hour sodium in (mg/day); Na spot = spot urinary sodium in mmol/L; Cr spot = spot urinary creatinine in mmol/L; PR Cr = Predicted 24-hour urinary creatinine (mg/d)].

The predicted 24-hour urinary creatinine was calculated using the following formula:

$PR\, Cr = 2.04 \times age (years) + 14.89 \times weight (kg) + 16.14 \times height (cm) - 2244.45$

The daily salt intake was estimated by multiplying 2.54 to the sodium excretion per day [10].

Statistical analysis

Data entry was done using Epicollect 5 (Imperial College London, London, UK) and analysis was done using SPSS version 19 (IBM Corp, Armonk, USA). Patients were divided into two groups - resistant and non-resistant hypertension. Frequencies with percentage were calculated for all categorical variables. Chi-square test was done to check the significance of the differences between the two groups. P-value < 0.05 was considered as statistically significant. Multivariable analysis (binary logistic regression) was done to identify the factors associated with RH. Those variables with a p-value < 0.2 in the unadjusted analysis were included in the multivariable model.

## Results

A total of 275 patients were included in the study. The mean age of the patients was 56 ± 10 years (range 28-81 years) and 61% (n=169) of the patients were females. The mean duration of hypertension was 7 ± 5 years (range 1-30 years). The mean BMI of the patients was 25 ± 4 kg/m2 and 77% (n=213) patients were overweight or obese. A family history of hypertension was present in 30% of patients (n=83) and 18% (n=50) patients had diabetes mellitus.

History suggestive of secondary hypertension was present in 36 (13%) patients of which eight (3%) patients had a history of recent weight gain, 30 (11%) patients had a history of sleep disturbances which include erratic sleep, reduced sleep (less than four hours), daytime sleepiness and snoring and 14 (5%) patients had a history of other drug intakes which can contribute to secondary hypertension. Left ventricular hypertrophy was present in 42% (n= 106) and hypertensive retinopathy was present in 40% (n=91). The most commonly used antihypertensive drugs were amlodipine (81%) followed by enalapril (53%) and thiazide diuretic (21%).

Blood pressure was controlled (< 130/80 mm of Hg) in 145 (53%) patients whereas, it was uncontrolled in 130 (47%) patients. Of 130 patients with uncontrolled hypertension, 81 (29%) patients had uncontrolled hypertension due to therapeutic inertia and 22 (8%) had uncontrolled hypertension due to non-adherence to drug therapy. Treatment-resistant hypertension was seen in 31 [(11%) 95% CI 8-16%] patients. Of these, 27 (10%) had uncontrolled resistant hypertension and four (1%) had controlled resistant hypertension (Table 1).

**Table 1:** Distribution of patients in various hypertension categories attending a tertiary care centre, Puducherry, south India, 2019 – 2020 Resistant hypertension includes both uncontrolled blood pressure on more than equal to three drugs or controlled blood pressure more than three drugs.

Category	Number	%
1. Controlled hypertension	145	53
On ≤ 3 drugs	141	52
On > 3 drugs (Resistant Hypertension)	4	1
2. Uncontrolled hypertension	130	47
Therapeutic inertia	81	29
Non-adherence	22	8
Resistant hypertension	27	10

There was a significant difference in the duration of hypertension, features of secondary hypertension, erratic sleep habits, body mass index (BMI), and fasting blood glucose (FBG) between patients with non-resistant and resistant hypertension (p-value ≤ 0.05) (Table 2).

**Table 2:** Factors associated with resistant hypertension

Variable	Non-resistant hypertension	Resistant hypertension	p-value
Age at Diagnosis			
< 40 years	31 (12.7%)	7 (22.6%)	0.28
40-59 years	178 (73%)	19 (61.3%)	
≥60 years	35 (14.3%)	5 (16.1%)	
Duration of hypertension			
≤5 years	117 (48%)	14(45.2%)	0.04
6-10 years	96(39.3%)	8(25.8%)	
>10 years	31(12.7%)	9(29%)	
Gender			
Male	98 (40.2%)	8(25.8%)	0.12
Female	146 (59.8%)	23(74.2%)	
Smoking			
Current smoker	6 (2.45%)	0	0.61
Former-smoker	21(8.6%)	2 (6.4%)	
Alcohol consumption	18 (7.37%)	0	0.12
Physical inactivity	74 (30.32%)	11(35.48%)	0.56
Family history of hypertension	72(29.50%)	11(35.48%)	0.49
Secondary drug intake	13(5.32%)	1(3.2%)	0.62
Features of secondary hypertension	26 (10.7%)	10(32.3%)	0.001
Disordered sleep	21(8.6%)	9(29%)	0.001
BMI			
Normal	60 (24.6%)	2(6.5%)	0.06
Overweight	62 (25.4%)	8(25.8%)	
Obese	122 (50%)	21(67.7%)	
24 hours Salt intake (mean)	3.06±0.54g/day	2.98±0.54 g/day	0.40
Fasting blood glucose			
<100 mg/dl	126(54.89%)	13(43.33%)	0.001
100-125 mg/dl	73 (31.06%)	4(13.33%)	
>125 mg/dl	36(15.31%)	13(43.33%)	
Serum creatinine ≤ 1.2 mg/dl > 1.2 mg/dl	194 (83.3%) 39 (16.7%)	25 (83.3%) 5 (16.7%)	0.99
Diabetes mellitus Yes No	41(16.8%) 203(83.19%)	9(29.03%) 22(7.09%)	0.09
Thyroid disorder Yes No	8(3.27%) 236(96.72%)	0 31(100%)	0.30
Dyslipidaemia Yes No	107(43.85%) 137(56.14%)	14(45.16%) 17(54.83%)	0.89

As the duration of hypertension increases, the prevalence of resistant hypertension increased. In the binary logistic regression analysis hypertension duration > 10 years, obesity (BMI ≥ 25), disordered sleep, and FBG> 125 were significantly associated with resistant hypertension (Table 3).

**Table 3:** Binary logistic regression analysis of factors associated with resistant hypertension FBS - fasting blood sugar

Variable	Adjusted OR (95% CI)	P-value
Sex		
Male	Reference	
Female	1.2 (0.5-3.1)	0.7
Duration of hypertension		
≤5 years	Reference	
6-10 years	0.5(0.2-1.5)	0.2
>10 years	3.4 (1.1-10.2)	0.03
BMI		
<22.9	Reference	
23-24.9	2.9 (1.0-7.7)	0.2
≥25	4.6 ( 1.2-16.8)	0.01
Diabetes Mellitus		
No	Reference	
Yes	0.8 (0.2-2.8)	0.7
Disordered sleep		
No	Reference	
Yes	4.1 (1.4-12.0)	0.01
FBS		
<100	Reference	
100-125	0.6 ( 0.2-2.2)	0.4
>125	5.7 ( 1.9-17.5)	0.002

## Discussion

Resistant hypertension increases end organ damage, cardiovascular morbidity, and premature death significantly [11]. We conducted a cross-sectional descriptive study to find out the prevalence of RH and the factors associated with it at a hypertension clinic in a tertiary care referral hospital from South India. The prevalence of RH in the present study was 11% and prevalence of pseudo resistance comprising of non-adherence to treatment and therapeutic inertia was 37%.

Therapeutic inertia is one of the important causes of uncontrolled hypertension [7]. It is defined as the failure of doctors to intensify medication regimens at encounters with patients who have an uncontrolled risk factor. Out of a total of 275 patients, 130 (47%) had uncontrolled hypertension in the present study. The most common cause of uncontrolled hypertension was therapeutic inertia 81/130 (62%), followed by nonadherence to medication 22/130 (17%). RH was responsible for uncontrolled hypertension in 21% (27/130) patients.

Duration of hypertension, elevated FBG, and disordered sleep was significantly associated with resistant hypertension. A history of erratic sleep was present in 29% of patients with RH. High salt intake causes uncontrolled hypertension but we could not demonstrate significant association between high salt intake and RH.

In a meta-analysis including 91 studies published between 1991 and 2017 with a pooled sample of 3,207,911 patients, the global prevalence of RH was 10.3% [5]. RH prevalence in neighbouring Pakistan was 12% [12] and in Sri Lanka it was 19% [7]. The definition of RH in these studies is based on the older target BP of < 140/90 mmHg. It is estimated that the prevalence of RH would increase by 4% if newly recommended target control of < 130/80 is considered [4].

In a study done in a secondary care setting in Jaipur, India, the prevalence of RH was found to be 19% [13], which is higher as compared to ours but this was a prescription based retrospective study, so accurate measurement of BP cannot be ascertained and also assessment of adherence to antihypertensive therapy was not done using validated scales. A similar prevalence of RH was found in another multi-centric study from India [14]. This study also had the above-mentioned limitations.

Even in the United States, the National Health and Nutrition Examination Survey (NHANES) prevalence estimates suggest that uncontrolled hypertension is a major issue with rates being as high as 51% in men and 45% for women overall and up to 30% among those who are on treatment [3]. High rates of therapeutic inertia like ours were also noted by Kumara et al in a Sri Lankan study [7]. Awareness among physicians regarding the accurate measurement of BP, regular monitoring, and titration of treatment regimens may improve the control of hypertension in the subcontinent.

Tomaszewsk et al reported higher rates of 25% of nonadherence among patients with uncontrolled hypertension from the UK [15]. Higher nonadherence rates in the latter study could be explained because adherence was assessed using high-performance liquid chromatography-tandem mass spectrometry (HP LC-MS/MS) urine analysis for 40 most commonly used hypertensive medication or their metabolites. It is also possible that since our institute provides free medicines during monthly follow-up visits, the adherence rate might be better in our patients.

Duration of hypertension, obesity, and elevated FBG were significantly associated with RH in this study. A similar association was also demonstrated in many other studies [4,7,12,13]. Disordered sleep also was strongly associated with RH in the present study. A case control study by Goncalves et al also showed a strong association between obstructive sleep apnea (OSA) and RH [16]. OSA causes intermittent hypoxia and sympathetic stimulation causing treatment-resistant Hypertension. In a follow-up study for five years in Spain, it was found that continuous positive airway pressure (CPAP) therapy in patients with OSA reduced the incidence of new-onset hypertension [17]. OSA is a modifiable risk factor of hypertension though it is highly unrecognized and untreated.

A Cochrane review of meta-analysis including 34 trials suggests a modest reduction of salt intake for a longer period of time causes a reduction in blood pressure. Salt intake of 4.4 gm/day on an average reduces BP 5/3 mm of hg in hypertensive and 2/1 mm of hg in normotensive people [18]. The gold standard method to measure daily salt intake is 24-hour urinary sodium excretion, though 24-hour urine sodium in patients with diuretics intake may not correlate with daily sodium intake. And 24-hour urine collection may not be accurate and practical. We have used spot urine sodium and creatinine to estimate 24-hour urinary sodium. World Health Organization recommends that salt intake for adults should be less than 5 grams per day and salt intake in all our study participants was lower than this recommendation.

To the best of our knowledge, this is the first study from South India on the prevalence of RH. We included an adequate sample size of 275 hypertensive patients attending a hypertension clinic. BP for all patients was measured by a single investigator (RM) with a mercury sphygmomanometer using standard precautions. We also systematically studied the adherence to medications using the Morisky Green Levine scale. However, our study had many limitations. The prevalence of resistant hypertension in our study (11%) might be an overestimation as we could not rule out white coat effect by 24-hour ambulatory BP measurement. It is possible that since we have separate clinics for cardiovascular diseases and kidney diseases, we had less number of patients with these comorbidities where prevalence of RH is found to be high [19]. We could not work up patients with RH to rule out secondary causes of hypertension and we could not do a 24-hour urinary collection for sodium excretion to estimate 24-hour salt intake.

## Conclusions

Prevalence of treatment-resistant hypertension in our study was 11% and longer duration of hypertension, obesity, history of disordered sleep, and elevated fasting blood glucose were associated with RH. Therapeutic inertia among doctors is very high which needs to be addressed with continued education and training.
